# Abnormal task driven neural oscillations in multiple sclerosis: A visuomotor MEG study

**DOI:** 10.1002/hbm.23531

**Published:** 2017-02-27

**Authors:** Eleanor L. Barratt, Prejaas K. Tewarie, Margareta A. Clarke, Emma L. Hall, Penny A. Gowland, Peter G. Morris, Susan T. Francis, Nikos Evangelou, Matthew J. Brookes

**Affiliations:** ^1^ Sir Peter Mansfield Imaging Centre, School of Physics and Astronomy, University of Nottingham Nottingham NG7 2RD United Kingdom; ^2^ Division of Clinical Neurology Queen's Medical Centre, University of Nottingham Nottingham NG7 2UH United Kingdom

**Keywords:** multiple sclerosis, post‐movement beta rebound, visual gamma, MEG, visuomotor abnormalities, neuronal oscillations

## Abstract

Multiple sclerosis (MS) is a debilitating disease commonly attributed to degradation of white matter myelin. Symptoms include fatigue, as well as problems associated with vision and movement. Although areas of demyelination in white matter are observed routinely in patients undergoing MRI scans, such measures are often a poor predictor of disease severity. For this reason, it is instructive to measure associated changes in brain function. Widespread white‐matter demyelination may lead to delays of propagation of neuronal activity, and with its excellent temporal resolution, magnetoencephalography can be used to probe such delays in controlled conditions (e.g., during a task). In healthy subjects, responses to visuomotor tasks are well documented: in motor cortex, movement elicits a localised decrease in the power of beta band oscillations (event‐related beta desynchronisation) followed by an increase above baseline on movement cessation (post‐movement beta rebound (PMBR)). In visual cortex, visual stimulation generates increased gamma oscillations. In this study, we use a visuomotor paradigm to measure these responses in MS patients and compare them to age‐ and gender‐matched healthy controls. We show a significant increase in the time‐to‐peak of the PMBR in patients which correlates significantly with the symbol digit modalities test: a measure of information processing speed. A significant decrease in the amplitude of visual gamma oscillations in patients is also seen. These findings highlight the potential value of electrophysiological imaging in generating a new understanding of visual disturbances and abnormal motor control in MS patients. *Hum Brain Mapp 38:2441–2453, 2017*. © **2017 Wiley Periodicals, Inc.**

## INTRODUCTION

Multiple sclerosis (MS) is an inflammatory, demyelinating and neurodegenerative disease of the central nervous system, affecting around 1 in 500 people (Mackenzie et al., 2014). Patients have a wide range of symptoms such as fatigue, problems with vision, limb movement, sensation and balance. Cognitive symptoms are common, with information processing speed being one of the first affected domains (Strober et al., 2009). Magnetic resonance imaging (MRI) is commonly used for diagnosis, and shows white matter lesions which are thought to affect around 60% of patients (Ormerod et al., 1986). However, white matter lesion load does not necessarily correlate with physical disability or cognitive impairment (Barkhof, 2002). This clinicoradiological paradox has led to the search for alternative verifiable markers of clinical status. Recent findings suggest that structural pathology goes well beyond classical white matter lesions, ranging from diffuse white matter damage (Kutzelnigg et al., 2005), to atrophy of the white and grey matter (Sanfilipo et al., 2006), including deep grey matter structures such as the thalamus (Houtchens et al., 2007) and cortical lesions (Sethi et al., 2013; Tallantyre et al., 2010). This suggests that disease status in MS is not the result of one structural deficit (i.e., white matter lesions), but rather results from a weighted combination of several types of structural pathology. It has been hypothesised that any type, or combination of structural damage will lead to disrupted brain function (Schoonheim et al., 2015). This implies that the characterisation of brain function and its disturbance in MS may prove key to determining the neural underpinnings of cognitive disturbances and physical disabilities. Neuroimaging investigations using techniques such as functional MRI (fMRI) and magnetoencephalography (MEG) are therefore of growing importance.

Previous fMRI studies have used a variety of tasks to determine the impact of demyelination on function, assessing visual, memory, and motor processes (Mainero et al., 2004; Reddy et al., 2000; Rocca et al., 2002), as well as cognitive function (Schoonheim et al., 2013a; Staffen et al., 2002). General findings suggest altered patterns of activation during attention and memory tasks (Mainero et al., 2004), a change in resting‐state activity (Liu et al., 2011), and disrupted connectivity (Dineen et al., 2009; Filippi and Rocca, 2013; Tewarie et al., 2015). However, findings from both task‐based and resting‐state studies have been somewhat contradictory (Schoonheim et al., 2015). In addition, a recent multimodal fMRI/MEG study has shown that MEG measures are more sensitive for detecting disease‐induced effects in cortical functional networks than fMRI (Tewarie et al., 2015), suggesting that MEG provides a good candidate to study perturbed brain function in MS patients.

MEG is a noninvasive method of assessing electrophysiological activity, via the measurement of extracranial magnetic fields generated by synchronised current flow in neuronal cell assemblies. Signals are dominated by oscillations resulting from the rhythmic activity of large clusters of neurons. Such neuronal oscillatory activity has been a topic of significant research in recent years, and many studies now show robust and focal changes in oscillations in response to simple tasks. For example, oscillatory changes during visuomotor tasks (visual and motor stimuli) have been well documented: in visual cortex, an increase in gamma (30–70 Hz) oscillations is observed in response to a visual stimulus (Adjamian et al., 2004; Brookes et al., 2005). Such gamma oscillations have been linked to a variety of behaviours such as visual perception (Melloni et al., 2007), attention (Fries et al., 2001), memory (Jensen et al., 2007), and motor control (Cheyne et al., 2008). Local field potential (LFP) recordings in animals support these findings, revealing stimulus induced gamma oscillations with varying amplitude and/or frequency depending on contrast (Henrie and Shapley, 2005), orientation (Friedman‐Hill et al., 2000), and velocity (Friedman‐Hill et al., 2000). In motor cortex, stimulus‐induced oscillatory changes are dominated by the beta band (13–30 Hz) which exhibits decreased power during movement (Jasper and Penfield, 1949) followed by a power increase (above baseline) on movement cessation (Neuper and Pfurtscheller, 2001). These phenomena are known as the event‐related beta desynchronisation (ERBD), and the post‐movement beta rebound (PMBR), respectively. Their origin is not understood fully; however, large beta amplitudes are believed to reflect inhibition (Cassim et al., 2001; Gaetz et al., 2011) and thus the ERBD might reflect an increase in local processing during motor activity, while the PMBR implies the inhibition of neuronal networks following activity (Alegre et al., 2008; Solis‐Escalante et al., 2012). Oscillations have also been closely associated with communication in the brain (Fries, 2005). Beta oscillations have been linked to resting‐state networks (Brookes et al., 2011a; Hipp et al., 2012; Siegel et al., 2012) and the PMBR in particular has been associated with long‐range connectivity in the sensorimotor system (Donner and Siegel, 2011; Liddle et al., 2016; Vidaurre et al., 2016). Similarly gamma oscillations have, for example, been related to feature binding in the visual system (Tallon‐Baudry and Bertrand, 1999). Such links between oscillations and connectivity suggest that oscillations themselves must depend heavily on the underlying (grey and white matter) myeloarchitecture (Hunt et al., 2016). It follows that such oscillations would likely be perturbed by the distributed myelin damage associated with MS.

To date, MEG has not been used widely in the study of MS, and the majority of studies have used resting‐state data (Cover et al., 2006; Hardmeier et al., 2012; Schoonheim et al., 2013b; Tewarie et al., 2013, 2014b; Van der Meer et al., 2013), with results generally showing a lower alpha peak frequency, decreased connectivity, and network disruption in patients (Tewarie et al., 2013, 2014a). Here, we use MEG to study MS patients whilst they undertake a visuomotor task. We test the hypothesis that the robust and well‐characterised neural oscillatory processes induced by this task will be perturbed significantly in patients compared to age‐ and gender‐matched healthy controls.

## METHODS

### Participants

The study was approved by the National Research Ethics Service and all participants gave written informed consent. MS patients (diagnosed with clinically definite MS (Polman et al., 2011) and no other known neurological conditions) were recruited from the Queen's Medical Centre in Nottingham, with healthy control subjects recruited to match the MS patient group based on age and gender. A total of 21 patients (12 females, age 42 ± 11 years (mean ± SD)) and 22 healthy controls (12 females, age 42 ± 12 years (mean ± SD)) were recruited (see Table 1 for further details).

**Table 1:** Descriptive variables for MS patients

	MS Patients (*n* = 21)	
	Mean	±SD
Age (years)	42	11
Disease duration (years)	7	6
EDSS	3 (median)	0‐6 (range)
MSSS	65	24
MS type		
RR	18	‐
PP	2	‐
SP	1	‐

EDSS = expanded disability status scale; MSSS = multiple sclerosis severity score; RR = relapsing–remitting; PP = primary progressive; SP = secondary progressive.

### Severity and Cognitive Scores

Disease severity was assessed by the Multiple Sclerosis Severity Score (MSSS) (Roxburgh et al., 2005) which determines disability progression in patients and is calculated based on disease duration and the expanded disability status scale (EDSS). All participants also undertook cognitive tests using the Brief International Cognitive Assessment for Multiple Sclerosis (BICAMS) (Langdon et al., 2012). This comprised the Symbol Digit Modalities Test (SDMT) (Smith, 2013), the California Verbal Learning Test (CVLT‐II) (Delis et al., 2000), and the Brief Visualspatial Memory Test – Revised (BVMT‐R) (Benedict et al., 1996). SDMT is a measure of information processing speed and involves participants pairing single digits with abstract symbols. The CVLT‐II provides a measure of verbal memory and requires participants to recall lists, while the BVMT‐R provides a measure of visual memory and requires participants to draw abstract shapes from memory. Patient cognitive scores were normalised using a *z*‐score taking age, gender, and education into consideration. Subjects also completed a Hospital Anxiety and Depression Scale (HADS) questionnaire.

### Task Paradigms

The visuomotor task comprised a centrally presented vertical square grating (angular field of view ∼7° both horizontally and vertically, spatial frequency = 3 cycles per degree, contrast = 100%) shown for 2 s followed by a 7 s fixation period during which participants viewed a centrally presented fixation cross. Participants were asked to press a button once, with their right index finger, in response to the appearance of the grating on the screen. The task consisted of 45 trials, lasting just under 7 min in total. In a separate experiment, resting‐state data were also acquired in the same subjects. Subjects were asked to lie still and ‘think of nothing' while MEG data were recorded continuously for 10 min. Patients were shown a fixation cross throughout the resting‐state scan. All MEG data were acquired with participants in a supine position.

### Data Acquisition

MEG data were acquired using a 275‐channel whole‐head CTF system (MISL, Coquitlam, Canada) operating in third‐order synthetic gradiometer configuration, at a sampling frequency of 600 Hz. Prior to acquisition, three localisation fiducial coils were placed on the head (at the nasion, and left and right preauricular points). These coils were energised periodically to continuously localise the position of the subject's head in the scanner. A 3D digitisation of the subject's head shape and fiducial locations were obtained using a 3D digitiser (polhemus Inc., Vermont) system. Subsequent co‐registration of the MEG data to an anatomical MRI (acquired using a 7 T Philips Achieva MRI scanner, or 3 T Philips Achieva for one participant, at 1 mm^3^ isotropic voxel resolution) was achieved via matching the digitised head surface to the equivalent surface extracted from the anatomical MRI.

### Data Analysis: Visuomotor Data

**Figure 1:**
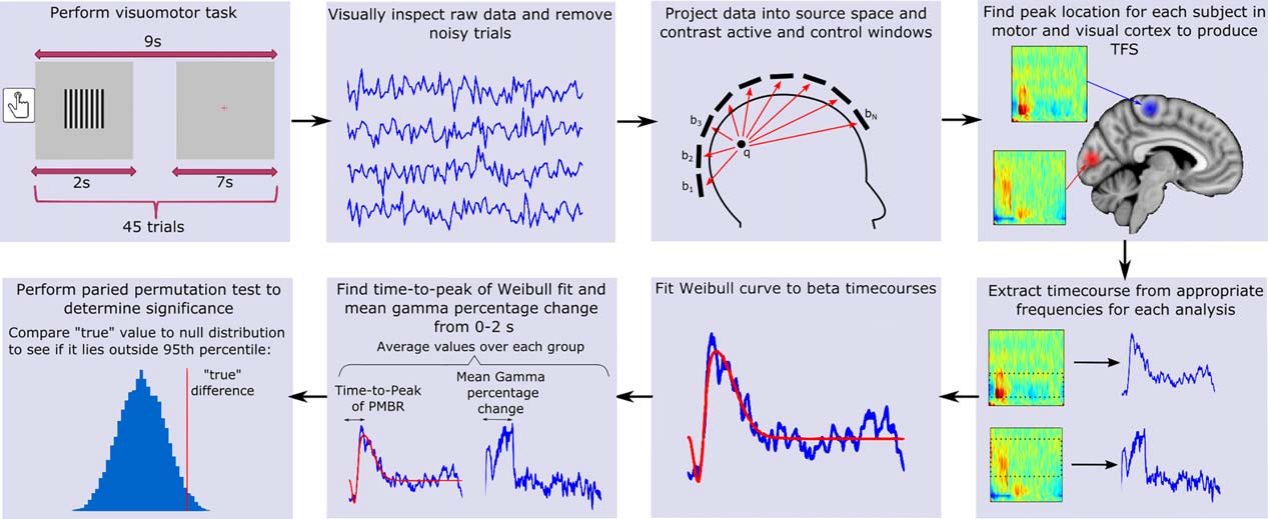
Schematic diagram summarising the stimulus and analysis methods for our visuomotor experiment. [Color figure can be viewed at http://wileyonlinelibrary.com]

An overview of our data processing methodology is shown in Figure 1. MEG data were inspected visually and noisy trials were removed. Trials with head movement >7 mm (Euclidean distance) from the starting position were also removed, leaving 43 ± 2 (mean ± SD) trials for healthy controls, and 42 ± 2 (mean ± SD) for MS patients (there was no significant difference between patient and control groups in the number of trials remaining). Lead fields were computed using a dipole approximation (Sarvas, 1987) and a multiple‐local‐spheres head model (Huang et al., 1999). A scalar beamformer (synthetic aperture magnetometry; Robinson and Vrba, 1998) was used to project MEG data into source space. Images showing gamma band power change were generated by contrasting 30–70 Hz power in an active window of 0–2 s, and a control window of 5–7 s, relative to stimulus onset. Images showing the spatial signature of task‐induced beta band power change were generated by contrasting oscillatory power in the 13–30 Hz band, using an ‘active’ window of 0–1 s and a ‘control’ window of 1–2 s relative to the button press. These windows were chosen to obtain the largest response by contrasting the ERBD to the PMBR. In a post‐hoc analysis, we also aimed to separate the ERBD and PMBR spatially; to this end, we contrasted a 0–0.5 s active window to a 6–6.5 s control window to assess the spatial location of ERBD. We further contrasted a 0.5–3 s active window to a 4–6.5 s control window to assess the spatial location of PMBR (all times relative to button press). In generating all the above images, to optimise spatial specificity, data covariance matrices used for beamformer weights calculation were generated using band‐limited data, averaged across active and control windows. Both our beta and gamma analysis produced pseudo‐*t*‐statistical images for each participant which were used to determine the peak location (location of the strongest response) of beta band power change in motor cortex during movement, and the peak increase in gamma oscillations in visual cortex during presentation of the grating. These locations, derived individually for each subject, were then used in subsequent analysis.

Timecourses measuring the evolution of electrical activity at each peak location (known as virtual sensor timecourses) were extracted, again using a scalar beamformer. Here, beamformer weights were calculated using a covariance matrix generated from the entire dataset, and frequency filtered in 1–150 Hz; this enabled derivation of time–frequency measurements spanning the whole trial length and showing task‐induced change in oscillatory amplitude in all frequency bands. Virtual sensor timecourses were filtered into 23 overlapping frequency bands in the range of 1–100 Hz. For each frequency band, a Hilbert transform was used to compute the amplitude envelope of oscillations, which was averaged across trials. Via concatenation of these envelope timecourses in the frequency dimension, a time–frequency spectrogram (TFS) was derived showing the percentage change in the Hilbert envelope of the oscillations in all frequency bands relative to a baseline; baseline was defined in the 5.8–6.9 s window relative to the button press for the motor response, and the 5–7 s window with respect to stimulus onset for the visual response. TFSs were averaged across participants in both groups.

TFSs were further analysed to test for differences between patients and controls in both the temporal signature of the movement induced beta band response and the magnitude of the visual gamma response. For visual gamma, the mean amplitude (in the 0–2 s time window and 30–70 Hz frequency range) was calculated for each group and the difference between groups was assessed. To test for differences in the timing of the beta rebound, the 13–30 Hz band was extracted from the TFS and modelled using a two‐parameter Weibull probability density distribution. This function has been shown previously to be highly suitable for modelling beta band timecourses (Liddle et al., 2016). The Weibull probability density distribution is given by

(1) $$f\left(t\right)=\frac{b}{a}{\left(\frac{t}{a}\right)}^{b-1}e^{-{\left(\frac{t}{a}\right)}^{b}},$$

where a is the scale parameter determining the width of the peak and b is the shape parameter that determines symmetry. Beta envelope modulation was modelled using two separate Weibull curves, one representing the ERBD and the other the PMBR. The ERBD was modelled using a Weibull function with a scale parameter ( a) ranging from 280 to 690 ms and a shape parameter ( b) of 3 (roughly symmetrical). The PMBR was modelled with a scale parameter ( a) of 500–2500 ms and a shape parameter ( b) ranging from 1 (negative skew) to 6 (positive skew). These parameters were chosen to provide a good fit to all subjects. These curves were entered as predictors into a series of general linear models for each participant's beta timecourse. The model with the best fit was chosen, and the time‐to‐peak of the rebound found. Fitting was applied to individual subjects and results averaged. Note that computing time‐to‐peak using the fit (rather than the data) reduces the effect of noise.

To determine statistical significance of the difference between healthy controls and MS patients, a paired permutation test was used: MS patients were matched with their healthy controls and the mean difference in the parameter of interest (gamma amplitude or beta time‐to‐peak), between all matched pairs was found. This was then repeated 20,000 times to generate a null distribution; on each iteration, the healthy control and patient in each pair were swapped randomly before recalculating the mean difference. In this way, we reasoned that if no genuine difference between groups was apparent, the labelling of the patient/control pairs would be meaningless and hence the genuine difference (with patients and controls labelled correctly) would fall inside the null distribution. The *P* value was determined by comparing the genuine value to the null distribution, assuming a two‐tailed test (i.e., time‐to‐peak of the beta response could be faster or slower in patients relative to healthy controls; similarly gamma amplitude in patients could be greater or less than matched healthy controls).

### Data Analysis: Resting‐State Data

The resting‐state data were used to test for differences in the resting amplitude of visual gamma and motor beta oscillations between groups. MEG data were inspected visually; noisy data segments and data segments with head movement >7 mm (Euclidean distance) from the starting position were removed. Segments were defined as contiguous 10 s windows; following visual inspection, 58 ± 3 (mean ± SD) segments remained for healthy controls and 55 ± 8 segments remained for MS patients (no significant difference). Regions of interest (ROIs) were generated in the left motor cortex and visual cortex for each subject. As above, a scalar beamformer was used to project data to these regions (using a covariance window spanning 1–150 Hz and a time window spanning the entire experiment). Following this, a Hilbert envelope was used to generate the mean (over all time) oscillatory amplitude across a range of frequencies between 1 and 100 Hz. These amplitude spectra were averaged over participants, and between group differences assessed.

## RESULTS

The task was well performed by all participants. Two MS patients had unusable MEG datasets caused by dental work and so these subjects and their paired healthy control subjects were removed from all analyses. Two patients were removed from the beta band analysis since no beta band response could be robustly identified, leaving a total of 17 MS patients (of which 2 were left‐handed, 2 were ambidextrous, and the remainder right‐handed) and 17 matched healthy control pairs (of which 1 was left‐handed, 1 was ambidextrous, 14 were right‐handed, and data were unavailable for 1) for the motor beta analysis. A single MS patient, whose gamma response lay more than 3 absolute deviations from the median of the MS group, was removed from the visual gamma analysis, leaving 18 MS patients and healthy controls. The raw cognitive tests compared matched pairs of 18 MS patients and healthy controls.

### Cognitive Tests

**Figure 2:**
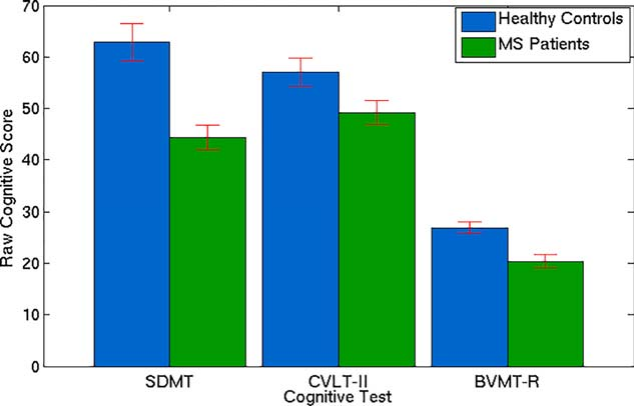
Raw cognitive scores for SDMT, CVLT‐II, and BVMT‐R tests for healthy controls (blue) and MS patients (green) with SE shown in red. Controls were found to score higher (significantly in SDMT and BVMT‐R) on all cognitive tests. [Color figure can be viewed at http://wileyonlinelibrary.com]

Figure 2 shows that the healthy control subjects performed significantly better than MS patients in 2 out of the 3 cognitive tests (SDMT: *P* < 0.0001, BVMT‐R: *P* < 0.0001), with a trend (CVLT‐II: *P* = 0.06) observed in the third. The SDMT score (healthy control score = 63 ± 4 (mean ± SE), MS patient score = 44 ± 2) is out of 102, the CVLT‐II score (healthy control score = 57 ± 3, MS patient score = 49 ± 2) is out of 80, and the BVMT‐R score (healthy control score = 27 ± 1, MS patient score = 20 ± 1) is out of 36. MS Patients were also found to be significantly more anxious and depressed on average compared to healthy controls (HADS: MS patient score = 16 ± 2, healthy control score = 9 ± 1, *P* = 0.009, maximum score possible is 42).

### Motor Response

**Figure 3:**
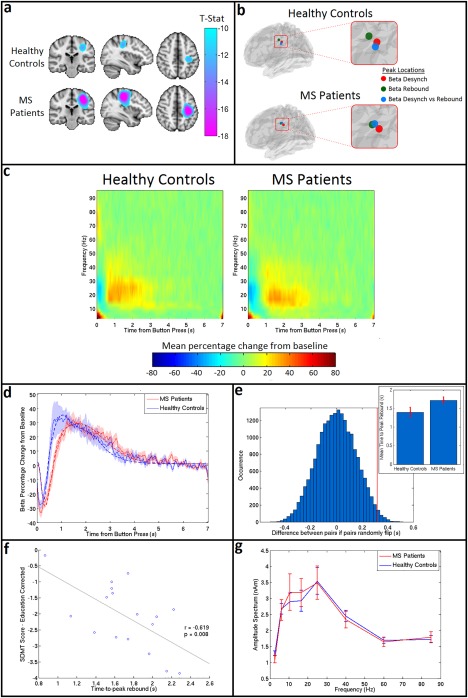
Beta band motor response. **(a)** Average *t*‐stat images for controls (top) and patients (bottom) contrasting 0–1 s to 1–2 s from button press in the beta (13–30 Hz) band. Images are shown on the same scale. **(b)** Average peak locations for healthy controls (top) and MS patients (bottom). The peak location for beta change is shown in blue, the location of maximum ERBD is shown in red, and the location of maximum PMBR in green. **(c)** TFSs for healthy controls (left) and MS patients (right) showing the mean percentage change in oscillatory power compared to baseline in response to a single button press. **(d)** The averaged beta response across all MS patients (red) and healthy controls (blue) with the SE shaded. The solid lines show the mean of the original responses, the dashed lines show the mean of the Weibull fits for each group. **(e)** A paired permutation test run on the rebound time for 20,000 iterations yielded a *P* value of 0.03 (two**‐**tailed test). The vertical red line shows the ‘true’ value in the difference between controls and patients, and blue shows the null distribution. Inset: the mean time‐to‐peak of the PMBR, measured using the Weibull fitting, with the SE shown on the error bars. **(f)** Correlation for MS patients between the time‐to‐peak of the rebound (*x*‐axis) and their corrected SDMT score (*y*‐axis). Plot shows a significant negative correlation (*P* = 0.008). **(g)** The amplitude spectrum for the resting state data (MS patients in red and healthy controls in blue) extracted from a region in left somatosensory cortex. Error bars show SE. [Color figure can be viewed at http://wileyonlinelibrary.com]

Results of the beta band analysis in sensorimotor cortex are shown in Figure 3. Figure 3a shows the beamformer images depicting the spatial location of maximum beta change (i.e. contrasting windows encapsulating ERBD and PMBR). Images are averaged over subjects within each group. Importantly, there is no significant difference in the spatial location of the peaks between groups (MNI co‐ordinates for healthy controls: [−37 ± 6, −28 ± 8, 41 ± 12] (mean ± SD), MNI co‐ordinates for MS patients [−36 ± 5, −26 ± 9, 47 ± 7]). Figure 3b shows the results of a post‐hoc analysis in which the peak location representing maximum beta change (shown here in blue) is contrasted with the peak location of ERBD and PMBR. In both groups, as would be expected from previous work (Fry et al., 2016; Jurkiewicz et al., 2006), a significant anterior shift in the location of PMBR relative to ERBD was observed. However, there was no significant difference in peak locations for either metric between groups. Figure 3c shows the TFSs, extracted from locations of interest (based on maximum beta change; i.e., the blue markers in Fig. 3b) in primary sensorimotor cortex. The TFS for MS patients is shown on the right and the TFS for healthy controls on the left. Note that, for both groups, the expected decrease in oscillatory power in the beta band immediately after the button press is apparent, and is followed by an increase above baseline on movement cessation. Further visual inspection suggests a lag in beta response time for MS patients compared to healthy controls. The mean beta responses and associated Weibull fits are shown in Figure 3d. The red curve shows the case for patients and the blue curve shows the case for controls. Note again the apparent PMBR delay in patients. Figure 3e shows the measured time difference alongside an empirically derived null distribution, with results indicating that the delay in time‐to‐peak of the PMBR is significant (*P* = 0.03; two‐tailed test). The measured time‐to‐peak of the beta response in both groups is shown in the inset image. It is noteworthy that the significant difference in time to peak of the PMBR occurs despite no measurable difference (*P* = 0.2) in the mean reaction time (RT) between controls (0.42 ± 0.03 s) and patients (0.46 ± 0.02 s). There was also no significant correlation in MS patients between reaction time and the time‐to‐peak of the PMBR (*P* = 0.3). Further post‐hoc analysis revealed that the significant difference in PMBR time‐to‐peak was also measurable at the peak location of ERBD (*P* = 0.04; two‐tailed test). The peak location of PMBR revealed the same pattern, but the effect failed to reach significance using a two‐tailed test. Figure 3f shows that, in MS patients, a significant negative correlation (*r* = −0.62, *P* = 0.008) exists between the time‐to‐peak of the PMBR and SDMT score, suggesting that patients who performed worse on the test had a longer delay in their peak rebound time. This relationship was not seen in healthy controls (*r* = −0.18, *P* = 0.48). (Note however that in controls, correlation was measured between time‐to‐peak and raw cognitive score, as a corrected score does not exist). No correlations were found between time‐to‐peak and any other MS severity or cognitive score. Finally, Figure 2g shows the amplitude spectrum of oscillations in sensorimotor cortex for resting‐state data acquired in the same subjects. There was no significant difference in the amplitude at any frequency.

### Visual Response

**Figure 4:**
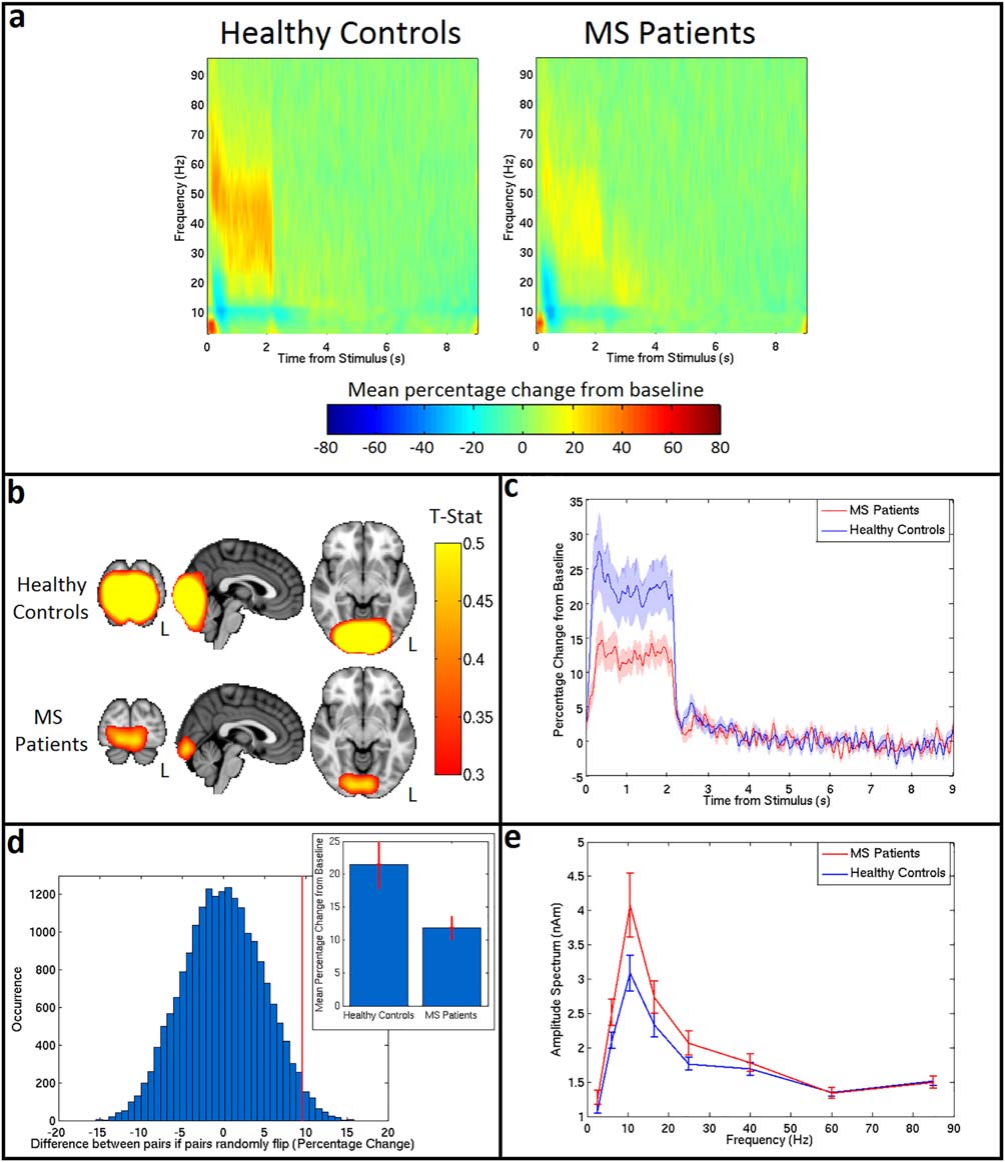
Visual gamma change. **(a)** TFS for controls (left) and patients (right) showing the mean percentage change in oscillatory power compared to baseline in visual cortex. **(b)** Average *t*‐stat images for controls (top) and patients (bottom) contrasting 0–2 s to 5–7 s from stimulus onset in the gamma (30–70 Hz) band. Images are shown on same scale. **(c)** Mean gamma (30–70 Hz) timecourses for healthy controls (blue) and MS patients (red) with the SE shown shaded. **(d)** A paired permutation test, yielding a *P* value of 0.04. The red line shows the ‘true’ mean difference between controls and patients, and blue shows the null distribution. Inset: mean percentage change in the gamma band (30–70 Hz) with SE shown in red. **(e)** The amplitude spectrum for resting state data (MS patients in red and healthy controls in blue) over a range of frequencies (for a location in the visual cortex) with SE shown on error bars. [Color figure can be viewed at http://wileyonlinelibrary.com]

Figure 4 shows the results of the gamma band analysis in visual cortex. Figure 4a shows the TFS plots for healthy controls (left) and MS patients (right), extracted from regions of interest in visual cortex. As expected, a strong increase in the gamma band activity is seen during stimulation for both groups. However, this response is diminished in MS patients. This finding is echoed in the *t*‐stat images in Figure 4b, which show a weaker response in MS patients. Plotting the timecourse of the mean gamma percentage change from baseline for patients (red) and controls (blue) (Fig. 4c) shows this clear reduction in the patient response. The increase in gamma power is significantly lower (*P* = 0.04) in patients than healthy controls, dropping from 21 ± 4% (mean ± SE) for healthy controls to 12 ± 2% (mean ± SE) for MS patients (see inset of Fig. 4d). This was demonstrated by a paired permutation test shown in Figure 4d; here the genuine difference between groups is shown by the red line, and the empirical null distribution is shown in blue. There were no measurable correlations between visual gamma power and cognitive, or illness severity scores. Finally, Figure 4e shows the amplitude spectrum for an ROI in visual cortex (MS patients in red, healthy controls in blue) during the resting state. No significant difference was seen at any frequency (although the apparent change in alpha oscillations is in some agreement with a previous finding (Van der Meer et al., 2013)) meaning that the significant effect of MS on gamma oscillations induced by visual stimulation occurs despite no effect on resting state gamma amplitude.

## DISCUSSION

Changes in MEG‐derived electrophysiological activity in MS patients have been documented previously (Cover et al., 2006; Hardmeier et al., 2012; Schoonheim et al., 2013b; Tewarie et al., 2013, 2014b; Van der Meer et al., 2013), but to date, relatively little work has been undertaken to study task‐induced changes. In this study, we measured the neural oscillatory response, in motor and visual cortices, in MS patients and matched healthy control subjects. Our results show that, although the characteristic motor and visual responses were present in patients, they were perturbed significantly relative to controls. Specifically, the time‐to‐peak of the PMBR was increased in MS patients; this was despite no measurable increase in reaction time, no correlation between reaction time and time‐to‐peak, and no difference in the amplitude of motor cortex beta oscillations in the resting state. Interestingly, the time‐to‐peak of the PMBR demonstrated a significant correlation (after multiple comparison correction) with the SDMT score (a measure of information processing speed), potentially suggesting a link to measurable behaviour. A significantly decreased gamma band response to visual stimulation was also observed and again this occurred despite a finding of no difference in resting‐state gamma amplitude in visual cortex. Overall these findings suggest that significant value can be found in measuring electrophysiological responses to visuomotor stimuli in MS patients. This might prove to be a useful avenue of research to characterise visual and motor deficiencies in MS.

The increased delay in PMBR in patients suggests a general slowing of the beta response. Given that this delay was observed in the absence of any measurable change in reaction time, it is likely that it represents a genuine pathophysiological aspect of the disease. Although observed robustly, the beta response during motor activity is not fully understood. A weight of literature suggests that beta oscillations are a marker of cortical inhibition. According to this theory, desynchronisation results from the activation of small patches of cortex serving some aspect of motor output, while the rebound is related to the return of those cortices to their original state (Pfurtscheller and Da Silva, 1999). This theory also emphasizes a network hypothesis, which suggests that the rebound is caused by active inhibitory input to the motor cortex (Gilbertson et al., 2005) which facilitates motor control by preventing further unwanted movements. This is supported by the fact that the PMBR does not develop until after childhood (Gaetz et al., 2010), an observation that might be related to the difficulty that young children experience in fine motor control. The inhibition theory is also supported by *in vivo* measurement and perturbation of neurochemistry. Neural oscillations are likely to reflect a balance between excitatory and inhibitory neurotransmission, which is mediated, in part, by glutamate and GABA, respectively. Previous findings using magnetic resonance spectroscopy (MRS) suggest a correlation between GABA and beta dynamics (Gaetz et al., 2011). Further, pharmacological manipulation of GABAergic inhibition also generates measurable changes in the beta envelope (Hall et al., 2010, 2011; Muthukumaraswamy et al., 2013). In this context, the perturbed PMBR in patients could relate to altered levels of GABAergic inhibition and this is supported by a finding of significantly lower GABA levels in sensorimotor cortex in MS patients (Cawley et al., 2015). A competing, but not mutually exclusive, hypothesis regarding PMBR is that it relates to long range communication between distal brain areas. Evidence for this has come largely from connectivity studies which show significant temporal correlation in the resting and task positive beta envelopes in left and right primary motor cortices (Brookes et al., 2011, 2011b; Hall et al., 2013), and also increased coherence during the PMBR following a self‐paced motor task (O'Neill et al., 2015; Vidaurre et al., 2016). Given this link to long‐range connectivity, coupled with (A) the well characterised demyelination in MS (Calabrese et al., 2010; Steenwijk et al., 2015), (B) the known role of myelin in neuronal conduction velocity (Goldman and Albus, 1968; Smith and Koles, 1970), and (C) the strong links between myeloarchitecture and neural dynamics and connectivity (Helbling et al., 2015; Hunt et al., 2016), it is unsurprising that the timing of the PMBR is delayed. Indeed we speculate that the reduction in white matter integrity, inherent to MS, could relate to a decreased ability to inhibit motor activity quickly, and hence explain why this process is slowed.

One of the main goals of functional imaging in MS is to generate biomarkers that relate significantly to cognitive decline in patients. Our demonstration that the time‐to‐peak of the PMBR in patients correlates negatively with the patient's corrected SDMT score is therefore of significant interest, particularly given that reduction in information processing speed has been shown to be the most affected cognitive domain in MS (Kail, 1998; Litvan et al., 1988; Strober et al., 2009). While the reason for this correlation is unclear, it could relate to the putative role of beta oscillations as a mechanism by which the brain facilitates a forward internal model of desired movement. For example, Tan et al. (2016) show that the amplitude of the PMBR correlates negatively with uncertainty in feedforward estimations. More generally, Cao and Hu (2016) suggest that the beta rebound might not only correlate with estimation uncertainty but also reflect an updating process of the forward model. Reduced information processing speed in MS patients, as indicated by the lower SDMT scores, would likely lead to increased uncertainty and error on the forward model; such effects may well drive the correlation between SDMT scores and the time to PMBR peak observed here. However, we should also point out that correlative findings in such small sample sizes should be interpreted cautiously; further verification of this relationship in a larger sample size is therefore critical before such links can be made. We also show that no relationship was found between the time‐to‐peak and physical disability. However, patients had a median EDSS score of 3, suggesting moderate disability of one “functional system,” for example, bladder, vision, and sensations (or multiple mild disabilities in a few systems), but no mobility issues. It could be for this reason that there is no correlation between the motor response and disability.

Gamma oscillations have been observed consistently using intracranial and noninvasive electrophysiological recordings. These oscillations are likely generated in pyramidal cell populations synchronized by GABAergic interneurons (Gonzalez‐Burgos and Lewis, 2012), and previous work (Buzsáki and Wang, 2012; Fries, 2009) suggests that such oscillations might offer a potential mechanism for information coding in the brain, particularly since gamma amplitudes have been consistently linked to task performance (Dickinson et al., 2015; Edden et al., 2009). In this study, MS patients were found to have a significant reduction in the amplitude of visual gamma oscillations in response to a black and white grating, compared with healthy controls. Vision problems are often experienced by MS patients. In fact, a study examining 2265 patients reported that 82% experience vision problems, with 41% reporting the impact of the problem as ‘moderate’ or ‘high’ (Hemmett et al., 2004). The most common problem with vision is optic neuritis where the optic nerve becomes inflamed. Visual evoked potentials have been found to be abnormal in individuals suffering from optic neuritis (Tobimatsu and Kato, 1998); however, to our knowledge, this is the first study to show the perturbed visual gamma oscillations in MS. The neurophysiological mechanism underlying this finding remains unclear, but could again relate to neurochemistry. Visual gamma oscillations have been related to GABA concentration (Muthukumaraswamy et al., 2009); for example, administration of the GABA uptake inhibitor Tiagabine has been shown to affect significantly the frequency of induced gamma oscillations (Magazzini et al., 2016). It follows that the present finding of decreased gamma amplitude in patients might relate to GABA abnormalities which have been previously shown in MS patients, albeit not directly in visual cortex (Cawley et al., 2015). Demyelination may also play a role in the perturbation of gamma oscillations; while beta band effects are thought to relate to long‐range communication, gamma oscillations have been suggested to be a more local phenomenon, generated in relatively small cortical neural networks (Donner and Siegel, 2011; Hall et al., 2014; Pfurtscheller and Da Silva, 1999; Zumer et al., 2010). This potentially suggests that demyelination of white matter tracts may play a lesser role in our gamma findings than beta findings. However, it is tempting to speculate that cortical demyelination in MS (Kutzelnigg et al., 2005; Tallantyre et al., 2010), coupled with a demonstrable link between cortical myelination and MEG networks (Hunt et al., 2016) might offer an explanation regarding the reduced gamma amplitude observed here. However, further work, potentially involving MRS, myelin mapping (Geades et al., 2016), and MEG is required to further elucidate this relationship.

Finally, there are a number of limitations of this study, primarily sample size; 18 patients were included in our visual gamma analysis and 17 in the beta band analysis. These sample sizes are small, as are the effect sizes that we describe, both the reduction in gamma amplitude and increased time‐to‐peak of the PMBR. It therefore follows that definitive conclusions regarding the effects that we observe can only be drawn following a repeated demonstration of the same effect in an independent cohort of MS patients. It is also important to be aware that the beta band response can be modulated by other factors. For example, Fry et al. (2016) show that PMBR is altered by force output. Similarly, the duration of movement may have an effect (Feingold et al., 2015). Unfortunately, in this study, we had no way of controlling for such effects; future studies of this nature should therefore employ paradigms with controlled isometric force outputs. Similarly our illness severity scores in patients did not relate directly to visual acuity and this may explain the reason why visual gamma amplitude failed to correlate with any metric of behaviour. Overall, a follow‐up study should aim to address more directly the relationship between patients’ motor and visual function, and the PMBR and visual gamma oscillations.

## CONCLUSION

MS is characterised by widespread structural damage, which likely leads to numerous functional deficits. In this article, we sought to characterise those functional deficits via assessment of the electrophysiological response to a simple visuomotor task. We found a significant increase in the time‐to‐peak of the PMBR in MS patients compared to healthy controls, and this correlates significantly (across patients) with a measure of information processing speed. A significant decrease in the amplitude of visual gamma oscillations in MS patients relative to healthy controls was also found. These electrophysiological imaging results offer a new insight into visual disturbances and abnormal motor control suffered by MS patients.
